# Childhood asthma and indoor allergens in Native Americans in New York

**DOI:** 10.1186/1476-069X-5-22

**Published:** 2006-07-21

**Authors:** Simona Surdu, Lupita D Montoya, Alice Tarbell, David O Carpenter

**Affiliations:** 1 Department of Epidemiology & Biostatistics, School of Public Health, University at Albany, SUNY, One University Place, Room 127, Rensselaer NY, 12144-3445, USA; 2 Department of Civil & Environmental Engineering, Rensselaer Polytechnic Institute, 110 8^th ^Street, MRC 315, Troy NY, 12180, USA; 3 Akwesasne Task Force on the Environment, Hogansburg NY 13655, USA; 4 Institute for Health and the Environment, University at Albany, SUNY, 5 University Place, A217, Rensselaer NY, 12144-3429, USA

## Abstract

**Background:**

The objective of this study was to assess the correlation between childhood asthma and potential risk factors, especially exposure to indoor allergens, in a Native American population.

**Methods:**

A case-control study of St. Regis Mohawk tribe children ages 2–14 years, 25 diagnosed with asthma and 25 controls was conducted. Exposure was assessed based on a personal interview and measurement of mite and cat allergens (*Der p 1*, *Fel d 1*) in indoor dust.

**Results:**

A non-significant increased risk of childhood asthma was associated with self-reported family history of asthma, childhood environmental tobacco smoke exposure, and air pollution. There was a significant protective effect of breastfeeding against current asthma in children less than 14 years (5.2 fold lower risk). About 80% of dust mite and 15% of cat allergen samples were above the threshold values for sensitization of 2 and 1 μg/g, respectively. The association between current asthma and exposure to dust mite and cat allergens was positive but not statistically significant.

**Conclusion:**

This research identified several potential indoor and outdoor risk factors for asthma in Mohawks homes, of which avoidance may reduce or delay the development of asthma in susceptible individuals.

## Background

Asthma is one of the most common chronic illnesses in the general population and is the most common chronic illness in children, with about 7.5 million children under age 18 in the United States having asthma in 2001 [1]. Asthma prevalence has been increasing to epidemic levels, especially in areas with high urbanization. Over 300 million people worldwide are affected by asthma, with a high negative impact on quality of life, productivity and health care costs [2]. Children aged 0 to 4 years show the largest increase in prevalence and incur greater health care use, while adolescents have the highest mortality. Peak asthma prevalence was 7.5% of the population in 1995. Lower socioeconomic status has been found to be related to increased rates of asthma morbidity and mortality, with asthma rates being consistently reported to be very high in minority populations and in people living in poverty [3,4]. This difference is reflected in the number of emergency room visits, hospitalizations and death, and is thought to reflect differences in risk factors of exposure and asthma control with socioeconomic status.

The risks for developing asthma are still uncertain, but depend on a complex interaction of hereditary and environmental factors. Risk factors that have been identified include: genetic predisposition (family history of atopy or asthma); perinatal factors (low birth weight, prematurity); allergen exposures (sensitization and exposure to cockroaches, house dust mites, rodents, furry animals and molds); infections (respiratory infections, especially those caused by respiratory syncytial virus); environmental air pollution; tobacco smoke; diet and obesity [5-13].

Some kinds of early life exposures, such as having older siblings, attending daycare, exposure to animals and being breastfed, have been reported to be protective, perhaps secondary to microbial factors and immunity [14-16]. People who have a diet rich in fruits and vegetables have a lower risk of respiratory disease, perhaps due to the antioxidant nutrients these foods contain [17,18].

House dust mites constitute an important indoor allergen (*Der p 1*). Exposure to dust mite allergens is a known risk factor for sensitization and is a trigger for asthma attacks [4]. Dust mites are ubiquitous in most humid and warm areas. Although 13 species of house dust mites have been identified, only three are common in homes: *Dermatophagoides farinae*, *Dermatophagoides pteronyssinus*, and *Euroglyphus maynei*. Most mite infestations in the United States are caused by *D farinae *or *D pteronyssinus *[19]. Dust mite exposure can trigger asthma exacerbations as well as contribute to the development of asthma [20]. The suggested threshold for sensitization to *Der p1 *is 2 μg/g house dust, with the threshold for exacerbation of symptoms being 10 μg/g [21].

Cats are currently the most popular pet present in the United States homes, with over 25% of all households having cats. The major cat allergen is *Fel d 1*, which causes IgE responses in >90% of people allergic to cats. Early exposure to pet dander may either be protective or result in sensitivity [22]. There is evidence that the effect of exposure to pets may be different in different relative risk groups, based on parental allergy. Cat allergen is present as small particles that can aerosolize and persist in the environment for months [22].

Reducing exposure to indoor allergens, especially in genetically susceptible children, can reduce the development of allergic sensitization to house dust mites and cat allergens, and this may prevent childhood asthma and decrease the frequency and severity of asthma attacks [23,24].

**Figure 1:**
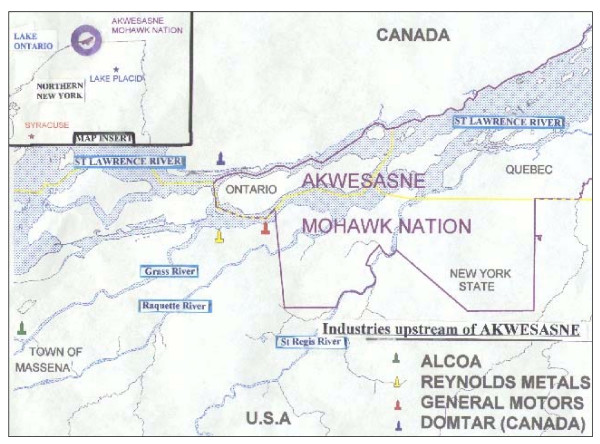
Map of the Akwesasne.

The Saint Regis Mohawk Tribe is a Native American nation located in northern New York State on both sides of the St. Lawrence River. The Saint Regis Mohawk Tribe Reservation (Akwesasne) contains lands in both New York State and Canada (Figure 1). The Akwesasne Mohawk Nation within New York State is located in Franklin County with a land area of 16.8 square miles, population of 2,558, and 945 housing units, according to 2000 U.S. census data. The Mohawk Territory of Akwesasne is adjacent to several sources of industrial pollution, including the General Motors Central Foundry Division, which is a National Priority Superfund Site, the Reynolds Metal Company and Aluminum Company of America, two New York State Superfund sites, as well as Domtar, a paper plant in Cornwall, Ontario. Several toxic substances (polychlorinated biphenyls [PCBs], polyaromatic hydrocarbons, fluorides, heavy metals) have been released from these industries over 25 years and have contaminated the air, soil, fish and wildlife in Akwesasne and the surrounding area. In analyses of the morbidity distribution and temporal trend of several chronic diseases among Mohawk people between the years 1992 and 1997, Negoita et al. [25] found a three-fold increased prevalence of asthma. While the annual incidence had a relatively small fluctuation from 1992 to 1996, the prevalence showed a very sharp increase as a result of new cases of childhood asthma. Exposure to PCBs [26] and dichlorodiphenyldichloroethene (DDE) [27] have been reported to increase risk of asthma. However, there was no evidence of an increase in environmental pollution, occupational exposure or tobacco use for the time period investigated. It is likely that the factors behind the increases in asthma prevalence among the Mohawks are similar to those elsewhere in developed countries [3,4], even though these are poorly understood.

The main objectives of this study were to evaluate the sources of indoor air pollution in the Mohawk community that may contribute to asthma in households at Akwesasne, to measure the levels of exposure to house dust mites and cat allergens, and to assess the correlation between potential risk factors and the childhood asthma.

## Methods

A case-control study was done on a population of 50 Mohawk children aged 2–14 years with and without asthma. The number of subjects was limited by the available funds allocated for this Pilot Study by the granting agency (EPA Region II). Sample analyses were completed as in-kind contribution by one of the authors (Dr. Montoya) so as to minimize lab expenses and maximize field expenditures. Of particular significance was the need to employ members of the Akwesasne community to conduct the field work, which constituted a large portion of the budget. Using the medical records at the St. Regis Mohawk Health Service, children 14 years of age and younger who were diagnosed with asthma were identified. Starting with those 14 years of age they were approached to participate in the study until a total of 25 asthmatic children had been recruited. Control subjects were also identified through the medical records of the St. Regis Mohawk Health Service based on lack of respiratory disease, and were selected by matching year of age and gender to the cases. Only one child per household was eligible to participate in the study, the oldest child being selected first. Informed consent was obtained from all participating children's parent or guardian. This study was approved by the University at Albany's Internal Review Board (human subjects assurance number FWA00001970, our protocol #99267).

The exposure information was collected through a personal interview and dust and air samples in the 50 Akwesasne households. The personal interview was conducted by a trained Mohawk interviewer and was given to both the child and a parent or caregiver. The questionnaire included information relevant to asthma risk factors, including: socioeconomic status (age, gender, marital status, educational level, employment status), family history of asthma, environmental tobacco smoke, the use of indoor combustion devices, the presence of biological contaminants, usage of pesticides, the presence of formaldehyde sources, pollution control methods, number of hours spent outdoors, smoking during pregnancy, birth weight and premature birth, breast-feeding history, and day-care attendance during the first two years of life. The last section of the interview asked about respiratory symptoms or signs known to be associated with indoor air pollution.

At the same home visit where the interview was obtained, dust and air sampling was performed. In each house two dust samples were collected, one from the living room (floor, rugs) and the other from the bedroom (floor, carpets, mattress, bedding), using a domestic vacuum equipped with a dust trap (The Boss Mighty Mite, Eureka, model 3670A, Bloomington, IN, USA). The air samples were collected using a filter cassette connected to a personal sampler (pump) running at 4.0 liters per minute. Since background concentration of airborne allergens are generally very low and they are usually detectable only during disturbances which cause the release of settled allergens, the filter was placed on the investigator collecting the dust sample at his/her breathing level while the dust sampling was performed. For allergen quantification of the mite and cat allergens indoor level (*Der p 1*, *Fel d 1*) in the dust and air samples, a two-site monoclonal enzyme-linked immunosorbent assay (ELISA) was used.

Data analyses were performed using SAS and Microsoft Excel. Sample data collected were assumed to be from a normally distributed population. As such, the means of the sample data were compared by Student's t-test. Allergen concentration data was transformed to a logarithmic scale with the assumption of normal distribution. The results were reported as geometric means and 90% Confidence Intervals for the means. Between group comparisons were conducted using log-transformed data. Proportions were compared by the Mantel-Haenszel Chi-Square test. Statistical significance was accepted at the 5% level.

## Results

The ages and gender of the Mohawk population studied is shown in Table 1. Controls were matched to cases by sex and year of age. Table 2 shows the prevalence and the risk of asthma associated to several self-reported hereditary and environmental factors assessed by the questionnaire. The risk of having asthma was elevated in the families with history of asthma (OR = 1.37), families where one member smoked in the home in the last 12 months (OR = 1.49) and during the lifetime of the child (OR = 1.18), having a mother who smoked during pregnancy (OR = 1.26), among children born prematurely (OR = 2.12), in families in which a garage was attached to the house (OR = 1.31) and families with a burn-barrel within a 5-min walk from the house (OR = 1.56). No relationship was found with moisture, mold, insects or pets in the home. This result may reflect the fact that the climate at Akwesasne is relatively dry and that it is too far north for cockroaches to be a major problem. The risk of having asthma was lower among the children who attended daycare (OR = 0.53). While none of these results were statistically significant, due to the small sample size, they are in the expected direction. The only statistical significant relationship found was for breast feeding that proved to be protective, with breast fed children having a 5.2-fold lower risk that those that were not (OR = 0.18, 90% CI = 0.05–0.75).

**Table 1:** Characteristics of the study population.

	**Number**	**Percent (%)**
Gender		
Boys	30	60
Girls	20	40
Age (years)		
2–6	16	32
7–10	14	28
11–14	20	40

**Table 2:** Prevalence and risks associated with asthma status for selected hereditary and environmental factors in the Mohawk population.

**Factor**	**Asthma cases with factor**			
	
	**No.^a^**	**%**	**OR**	**90% CI**
Family history of asthma	11	84.6	1.37	0.15–12.81
Smoking in the home in the last 12 months	8	32.0	1.49	0.52–4.23
Smoking in the home during the child's lifetime	11	45.8	1.18	0.45–3.09
Garage attached to the home	5	20.0	1.31	0.39–4.43
Burn-barrel within 5-minute walk from the home	19	79.2	1.56	0.52–4.74
Pet(s) in the house	11	44.0	0.73	0.29–1.85
Moist walls, ceilings, carpets, furniture	5	20.0	0.95	0.30–3.05
Mold in the house	8	36.4	0.83	0.30–2.29
Cockroaches, ants, other insects in the house	13	52.0	0.77	0.30–1.99
Smoking during pregnancy	6	24.0	1.26	0.41–3.90
Born before due date	8	32.0	2.12	0.67–6.69
Breast feed infant	17	68.0	***0.18***	0.05–0.75*
Attended day-care during the first two years of life	5	20.0	0.53	0.18–1.57

^a ^Total number of asthma cases differ because of missing data

* Mantel-Haenszel Chi-Square p-value = 0.0357

The results of mite and cat allergen measurements are summarized in Table 3. The cat allergen level (*Fel d 1*) was measured in 47 living room and bedroom dust samples, of which 2 bedroom samples were below the detection limit. The mite allergen (*Der p 1*) analyses were performed in 22 dust samples collected from the living rooms and bedrooms. Mite allergen levels were below the detection limit in 2 living room dust samples and 1 bedroom dust sample. The allergens found ranged from non-detectable levels to high levels of exposure that could be associated with asthma symptoms and severity. *Fel d 1 *levels ranged between 0.05 to 10.02 μg/g dust, with a geometric mean of 0.26 μg/g dust in the living room samples and between 0.02 to 12.64 μg/g dust, with a geometric mean of 0.26 μg/g dust in the bedroom samples. *Der p 1 *levels ranged between 0.05 to 11.15 μg/g dust, with a geometric mean of 0.25 μg/g dust in the living room samples and between 0.05 to 10.52 μg/g dust, with a geometric mean of 0.38 μg/g dust in the bedroom samples. The bedroom dust samples had a lower concentration of cat allergen (*Fel d 1*) and higher concentration of mite allergen (*Der p 1*) as compared to the living room dust samples, but the differences were not statistically significant. The dust allergen measurements showed a strong positive correlation between the *Der p 1 *levels found in the living room and in the bedroom (Bonferroni-adjusted significance level of 0.001).

**Table 3:** Concentration of major mite and cat allergens in living room and bedroom dust samples collected from Akwesasne households.

	**No. samples analyzed**	**No. samples with detectable levels**	**Allergen concentration (μg/g dust)**	
			
			**Geometric Mean**	**Range**
Cat allergen (*Fel d 1*)				
Living room	47	47	0.26	0.05–10.02
Bedroom	45	43	0.19	0.02–12.64
Mite allergen (*Der p1*)				
Living room	22	20	0.25	0.05–11.15
Bedroom	22	21	0.38	0.05–10.52

The air sampling did not yield any samples with detectable levels of either allergen. This was due primarily to the small volume of air sampled. With the exception of cat allergen levels measured in the living room, dust samples of all the allergen concentration means were higher in the group of children with asthma (Table 4). The mean concentration of *Der p 1 *in the households of children with asthma was twice as high as in the households of the control group, but the number of dust samples analyzed for the control group was too small to demonstrate statistical significance for the observed difference.

**Table 4:** Concentration of major mite and cat allergens in dust samples collected from Akwesasne households by site and case/control group.

	**Living room samples concentration (μg/g dust)**			**Bedroom samples concentration (μg/g dust)**		
	
	**No. of samples**	**Geometric Mean**	**90% CI**	**No. of samples**	**Geometric Mean**	**90% CI**
Cat allergen (*Fel d 1*)						
Asthma cases	23	0.21	0.12–0.36	22	0.22	0.12–0.42
Controls	24	0.31	0.20–0.49	23	0.17	0.09–0.33
Mite allergen (*Der p 1*)						
Asthma cases	17	0.29	0.12–0.69	17	0.43	0.19–0.93
Controls	3	0.12	0.01–1.75	4	0.23	0.03–1.84

Dust mite and cat allergen levels were not found to be statistically different in the group of subjects with asthma as compared to the subjects without asthma, neither in the living room dust samples nor in the bedroom dust samples.

**Figure 2:**
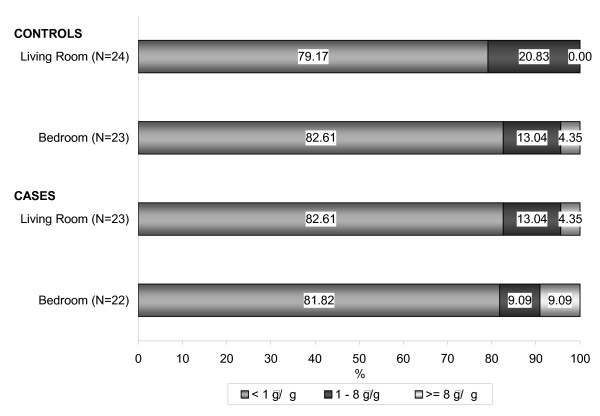
Frequency distribution of cat allergen levels (*Fel d 1*) in dust samples collected in homes of Mohawk children.

The association between allergens and asthma was also analyzed by using the cut off values of 1 and 8 μg/g of cat allergen per gram of dust. For cat allergens, any levels in the range of 1–8 μg/g of *Fel d 1 *per gram of dust is considered to pose a significant risk for sensitization. Levels above 8 μg/g of *Fel d 1 *per gram of dust are reported to be associated with acute asthma symptoms and severity [8]. Figure 2 illustrates the frequency distribution of cat allergen levels in the dust samples collected from the households of children with asthma. The results of Mantel-Haenszel Chi-Square test showed no significant association between *Fel d 1 *levels measured and asthma status in our study population.

**Figure 3:**
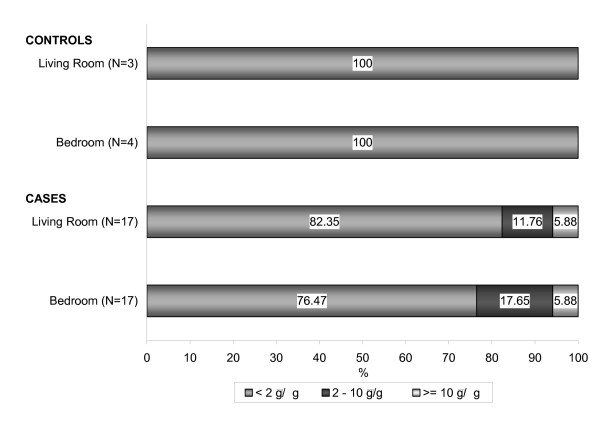
Frequency distribution of dust mite allergen levels (*Der p 1*) found in homes of Mohawk children.

The level of 2 μg/g of dust mite allergen (*Der p 1*) is the generally recognized threshold for sensitization and symptom development among susceptible individuals and 10 μg/g is reported as a risk factor for acute symptoms. These levels have been used to categorize the mite allergen levels in three groups and the frequency distribution in the asthma group are presented in Figure 3. The results of statistical correlation analysis between the dust mite allergen concentration levels and asthma status were not statistically significant.

The mite and cat allergen levels measured in living room and bedroom dust samples were combined in one household variable of exposure per allergen type. The dust mite allergen concentrations measured in the living room have been found to be significantly positive correlated with those measured in the bedroom, with a higher mean value in the bedroom. A subject was classified to be exposed to sensitization or asthma trigger levels if he/she was exposed to such levels in at least one of the house rooms. The asthma risk analyses results presented in Table 5 showed a positive association between current asthma and exposure to cat allergen (*Fel d 1*) above 8 μg/g dust in living room and/or bedroom of 3.45 and a population attributable risk of 9.3%, but with no statistical significance due to the small sample size. When using 1 μg *Fel d 1 *per g dust as the cut off level for cat allergen exposure, 30.4% of children with asthma and 29.2% of children without asthma were exposed and a small, but not statistically significant, positive association was found (OR = 1.06, PAR = 1.8%). The odds ratio for the association between exposure levels to indoor dust mites and current asthma could not be calculated since all the children without asthma were classified as non-exposed subjects. Three children (2 boys, ages 5 and 8; 1 girl, age 14) were exposed to sensitization or asthma trigger levels to both allergens, *Fel d 1 *and *Der p1*, and all three were diagnosed as asthma cases.

**Table 5:** Prevalence of house dust mite and cat allergen exposure by current asthma status in the Mohawk study population.

**Allergen level**	**Asthma subjects**		**Control subjects**		**OR**	**Population Attributable Risk (PAR)**
			
	**No.**	**Exposed**	**No.**	**Exposed**		
*Fel d 1*						
≥ 8 μg/g dust	23	13.0%	24	4.2%	3.45	9.3%
≥ 1 μg/g dust	23	30.4%	24	29.2%	1.06	1.8%
*Der p 1*						
≥ 10 μg/g dust	17	11.8%	4	0%	-	11.8%
≥ 2 μg/g dust	17	29.4%	4	0%	-	29.4%

## Discussion

The results of the present study are consistent with the evidence that premature children are at an increased risk of asthma, the risk being increased two-fold for the subjects born prematurely [5,6]. Previous studies have suggested that the development of protective immune responses to microbes early in life may reduce the likelihood of the development of allergies, and as a result the development of asthma [14], which would be in accordance to our findings related to daycare attendance as protective factors for current asthma.

Different studies indicate that asthma morbidity among Native Americans/Alaskan Natives is very common and may be higher than other racial/ethnic groups [25,29-34]. According to the 2001 California Health Interview Survey [35], Native Americans were more likely to have been diagnosed with asthma than any other racial/ethnic group. Approximately 25.5% of Native American children and 20% of Native American adults have been diagnosed with asthma in their lifetime, compared to 21% of African American children and 16% of African American adults, 14% Caucasian children and adults, 11.7% Asian children and 9.2% Asian adults, and 10% of Hispanic children and 7% of Hispanic adults.

According to the New York State Department of Health [36], the three year average (2000–2002) asthma discharge from hospitalization rates per 10,000 population for children under 14 years of age living in the Akwesasne area was 21.4, which is much higher than in the adjacent Franklin County rate of 13.3 and higher than the New York State rate (excluding NYC) of 18.1. The U.S. Census 2000 Demographic Profile showed that the total population in the New York portion of Akwesasne was 2,558 inhabitants, of which 96.5% are Native American. The population age distribution indicated that 762 inhabitants were between 0–14 years old. This indicates the difficulties of performing this type of study on such a small population.

The major limitation of our report is the size of the population studied. Because the population available for investigation was small, few of the results were statistically significant, although most of the results were in the direction expected from previous studies. We found an increased risk of current asthma in children associated with a self-reported family history of asthma, current and lifetime childhood environmental tobacco smoke (ETS) exposure, and air pollution (garage attached to the house, a burn-barrel within 5-min walk from the house), but none of these results were statistically significant. This problem is difficult to avoid when one studies a small population. Since attention to quantitative estimates of house allergen exposure in relation with asthma in children has received relatively little attention, we believe our results to be useful in spite of this problem. This is particularly true for Native American children in rural communities.

The Institute of Medicine [4] concluded that there is sufficient evidence to associate environmental tobacco smoke exposure with the development and exacerbation of asthma. Maternal smoking during pregnancy increased the occurrence of physician-diagnosed asthma during childhood in our study population, a finding which is consistent with the literature suggesting that the exposure to tobacco compromises the development of the fetal lungs and as a result, an increased risk of developing asthma [9,37].

Perinatal factors, including gestational age and birth weight, influence the development of atopy in early life, increase the risk of developing lower respiratory tract infections, and play a possible role in the development of asthma in later life [5,6]. Human milk contains numerous components protecting the infant against infections, including factors that provide specific immunity, nonspecific protective factors that inhibit the binding of bacterial pathogens and their toxins, and lipids that may disrupt enveloped viruses [38]. Breast milk contains cytokines and growth factors that may play an important role in modulating the development of asthma by preventing sensitization to environmental allergens, enhancing infant lung development and reducing susceptibility to respiratory infections [38,39]. The role of breastfeeding as a protective factor against asthma and atopic diseases, however, continues to be controversial, with some studies showing a negative effect of breastfeeding [40,41], and others showing a protective association [16,42,43]. There are, however, differences among various studies in relation to breastfeeding definition, study populations and age of the child when the association is assessed. Immunoprotection conferred by human milk may vary in relation with the mother's atopic constitution, infections, immune status, and diet. Our results support a strong protective effect of breastfeeding against current asthma in children under 14 years of age.

The levels of dust allergens measured in the Akwesasne households ranged from non-detectable to values exceeding the sensitizing or asthma symptoms triggering threshold concentrations. The threshold values for allergen exposure most widely accepted are as follows: 2 μg or more of Group 1 mite allergens per gram of dust (*Der p 1, Der f 1*) as a risk factor for development of IgE antibody and asthma, and a higher level of 10 μg Group 1 mite allergens per gram of dust as a risk factor for an acute attack of asthma; 1 μg or more of cat allergen (*Fel d 1*) per gram of dust as a risk factor for sensitization to cats, and a higher level of 8 μg cat allergen per gram of dust as a risk factor for asthma symptoms in most cat allergic patients [21,28].

Dust mite allergen levels reported by other studies conducted around the world are characterized by high variability, dependent mostly upon humidity levels, from less than 2 μg/g of dust in very cold or dry climates (e.g., Stockholm, Arizona) to mean levels in the range of 2–15 μg/g in countries with climates more suited to mite reproduction (costal areas of Europe and USA). In some regions of Australia, Singapore and South America where the climate is suited for mite growth throughout most of the year, the mean allergen levels range between 10–40 μg/g [41]. The dust mite allergen levels found in the Akwesasne area ranged between 0.05–11.15 μg/g, and approximately 80% of the households had levels less than 2 μg/g. The Akwesasne Reservation is situated on the 45^th ^parallel of latitude with fairly moderate climate that puts our results in line with the findings published in the literature.

Pets are one of the most common indoor allergens along with dust mites, cockroaches and molds. In many countries, over 50% of homes have cats and/or dogs [44]. In our study population, 48% of the Mohawk households had pets in the house, and 22% of the study subjects reported having one or more cats in the home. The mean value of cat allergen measured in dust samples ranged between 0.02–12.64 μg/g, and about 18% of the households had levels over 1 μg/g that is the suggested threshold for sensitization. The cat allergen results showed a higher mean value in the dust samples collected from the living room as compared to the bedroom, which is in concordance with the results of other studies of cat allergen distribution, possibly due to the fact that pets are not allowed in bedrooms.

It is difficult to compare the dust mite and cat allergen levels between studies due to different study populations and dust sampling settings and methods (e.g., vacuum cleaner used, filter size, time and area sampling). Furthermore, the dust mite and cat allergen concentrations vary greatly by indoor humidity and consequently by climate, season, building floor, ventilation rates, dampness, floor type, occupant density, and many other factors indirectly associated with humidity.

The dust mite mean levels in our study were higher in the samples collected from households of children with current asthma, and this was also true for the cat allergen measured in bedroom samples. The mite allergen mean value was about two times higher in asthmatic children than controls, but the difference was not statistically significant due to the small baseline sample size. There was a slight increase in the risk of asthma in children living in homes with cat allergen concentrations over the sensitizing value of 1 μg/g (OR = 1.06; PAR = 1.8%) and a three-fold increase in risk of asthma for children exposed to 8 μg/g or more (OR = 3.45; PAR = 9.3%). Thirteen percent of children with current asthma had cat allergen exposure over 8 μg/g as compared to only 4.2% of non-asthmatic children.

The results reported by other case-control studies showed either a small positive or negative association between allergen exposure and current asthma in children. A case-control study conducted on 126 children, 1–15 years of age, found a small positive association of *Fel d 1 *over 8 μg/g and current asthma (OR = 1.4; PAR = 13%) [45], similar to those showed by another study conducted on 97 children, 12–14 years of age, for exposure over 10 μg/g (OR = 1.4, 1.6; PAR = 17%, 21%) [46,47]. Another case-control study conducted on 57 children, 3–15 years of age, found a negative association between cat allergen exposure over 10 μg/g and current asthma [48], as well as in the previously mentioned study on children 12–14 years of age exposed to levels over 2 μg/g. The risk of current asthma in relation to dust mite allergen exposure could not be calculated due to the fact that all control subjects were free of sensitizing or triggering exposure. All of these studies, including ours, are limited by the fact that the number of analyzed samples was small.

## Conclusion

Asthma has been considered rare among Native Americans, but in the last 20 years asthma prevalence is increasing, especially in children. Mohawk children are at risk of exposure to a number of perinatal and environmental factors which may be causally related to the increasing rates of asthma in this population. While most results did not show statistically significant differences due to the unavoidably small sample size, Mohawk households of children with current asthma had higher mean values of cat allergen (*Fel d 1*) and dust mite allergen (*Der p 1*) as compared to homes of children without the disease. The risk of current asthma was increased 3.5 fold for children exposed to cat allergen in dust above the triggering threshold level. All of the children without asthma had dust mite allergen concentrations under the sensitizing threshold, while over two-thirds of children with current asthma were above this concentration. Breast feeding was found to be a significant protective factor against asthma. These observations indicate that asthma is not only a problem for inner city minority populations, but also is an important public health issue in rural Native American communities. Further study of indoor and outdoor risk factors which trigger asthma attacks and study of means to reduce or delay the development of asthma in susceptible individuals in this population are needed.

## Competing interests

The author(s) declare that they have no competing interests.

## Authors' contributions

SS participated in the planning of the study, carried out the data analysis and wrote the first draft of the manuscript. LM assisted in the planning of the study, trained both AT in the sample collection and performed allergen measurements. AT, who lives at Akwesasne, collected all of the samples and sent them to Albany. DC supervised the overall project, provide fiscal and administrative management and prepared the final manuscript for submission for publication. All authors read and approved the final manuscript.
